# High Throughput Measurement of Locomotor Sensitization to Volatilized Cocaine in *Drosophila melanogaster*

**DOI:** 10.3389/fnmol.2018.00025

**Published:** 2018-02-05

**Authors:** Ana Filošević, Sabina Al-samarai, Rozi Andretić Waldowski

**Affiliations:** Department of Biotechnology, University of Rijeka, Rijeka, Croatia

**Keywords:** neuronal plasticity, cocaine, circadian genes, locomotor activity, locomotor sensitization, *Drosophila melanogaster*, addiction

## Abstract

*Drosophila melanogaster* can be used to identify genes with novel functional roles in neuronal plasticity induced by repeated consumption of addictive drugs. Behavioral sensitization is a relatively simple behavioral output of plastic changes that occur in the brain after repeated exposures to drugs of abuse. The development of screening procedures for genes that control behavioral sensitization has stalled due to a lack of high-throughput behavioral tests that can be used in genetically tractable organism, such as *Drosophila*. We have developed a new behavioral test, FlyBong, which combines delivery of volatilized cocaine (vCOC) to individually housed flies with objective quantification of their locomotor activity. There are two main advantages of FlyBong: it is high-throughput and it allows for comparisons of locomotor activity of individual flies before and after single or multiple exposures. At the population level, exposure to vCOC leads to transient and concentration-dependent increase in locomotor activity, representing sensitivity to an acute dose. A second exposure leads to further increase in locomotion, representing locomotor sensitization. We validate FlyBong by showing that locomotor sensitization at either the population or individual level is absent in the mutants for circadian genes *period (per)*, *Clock (Clk)*, and *cycle (cyc)*. The locomotor sensitization that is present in *timeless (tim)* and *pigment dispersing factor (pdf)* mutant flies is in large part not cocaine specific, but derived from increased sensitivity to warm air. Circadian genes are not only integral part of the neural mechanism that is required for development of locomotor sensitization, but in addition, they modulate the intensity of locomotor sensitization as a function of the time of day. Motor-activating effects of cocaine are sexually dimorphic and require a functional dopaminergic transporter. FlyBong is a new and improved method for inducing and measuring locomotor sensitization to cocaine in individual *Drosophila*. Because of its high-throughput nature, FlyBong can be used in genetic screens or in selection experiments aimed at the unbiased identification of functional genes involved in acute or chronic effects of volatilized psychoactive substances.

## Introduction

Behavioral sensitization is a relatively simple change in behavior, which is induced by repeated exposure to drugs of abuse. It is studied in laboratory animals as a behavioral endophenotype that aids in dissection of mechanisms of neuromodulation that are triggered by drug administration. It is primarily, but not exclusively, studied in relation to the motor-activating effects of the psychostimulants cocaine and methamphetamine (Steketee and Kalivas, 2011; Marinho et al., 2015). Sensitization following repeated administration of psychostimulants in rodents can be quantified as an increase in the amount of locomotion (locomotor sensitization), or an increase in the frequency or intensity of other, often stereotypical, motor behaviors such as rearing (behavioral sensitization).

The discovery that *Drosophila melanogaster* can develop behavioral sensitization to cocaine provided new genetic targets for testing of this phenomenon in vertebrate model organisms (McClung and Hirsh, 1998, 1999). Behavioral sensitization in *Drosophila* is regulated by the circadian genes *period (per)*, *Clock (Clk)*, *cycle (cyc)*, and *doubletime (dbt)*, which provided at the time the first demonstration that circadian genes modulate drug-induced plasticity (Andretic et al., 1999). Connection between a drug-induced behavior and the circadian system was further demonstrated by identification of *Lmo* (LIM-only) gene in a genetic screen for cocaine sensitivity in *Drosophila* (Tsai et al., 2004). Studies in rodents confirmed the importance of these genes in the regulation of multiple behavioral and cellular addiction-related phenotypes (Abarca et al., 2002; McClung et al., 2005; Lasek et al., 2010; Parekh et al., 2015).

Sensitization of *Drosophila* to cocaine has several direct parallels with behavioral sensitization in mammals, including: persistence over time, sexual dimorphism and conservation of both molecules and mechanisms (Andretic et al., 1999; McClung and Hirsh, 1999; Bainton et al., 2000; Park et al., 2000; Nestler, 2004; Chen et al., 2009). Dopaminergic signaling was shown to be important for the acute motor-activating effects of cocaine, ethanol and nicotine in *Drosophila* (Bainton et al., 2000; Kong et al., 2010), consistent with presynaptic modulation of dopamine and serotonin neurons being important for the development of behavioral sensitization to cocaine (Li et al., 2000). Cloning and characterization of cocaine-sensitive fly dopamine transporter (dDAT) provided a concrete cocaine target on which to base genetic study of the neurophysiology of drug-induced effects in the brain (Porzgen et al., 2001).

In order to identify genes functionally involved in the modulation of brain functions, the ideal approach is to use a genetic screen in a genetically tractable model organism. However, the classic method used for induction of behavioral sensitization in *Drosophila* proved inadequate for use in a genetic screen, due to variability arising during drug delivery and the behavioral scoring procedure (McClung and Hirsh, 1998). This procedure was also time consuming because it required visual analysis of each individual fly’s behavior and assignment of a score based on a descriptive scale.

To decrease the variability during delivery, two new methods were developed that each delivered precise drug concentration to individual flies. In one, abdominal injections were paired with measurement of the locomotor activity using a commercially available monitoring system (Dimitrijevic et al., 2004). This demonstrated that sensitization can be induced via repeated injections and can objectively be measured as an increase in the forward locomotion. However, cocaine injections where time–consuming and required complex animal handling. The second method successfully decreased variability by delivering precise amounts of drug onto the fly’s cuticle using an airbrush (Lease and Hirsh, 2005). This approach revealed that the nervous system of flies is sensitive to topical drug application; however, scoring still had to be done using the descriptive scale. Ultimately, both methods required complicated animal handling and non-standard instrumentation, which rendered them inadequate for use in a behavioral screen.

Attempts to objectively quantify the scoring procedure were done by taking advantage of negative geotaxis, that is the fly’s ability to climb vertical surface (Bainton et al., 2000; George et al., 2005). Cocaine administration can, depending on dosage, either partially or fully affect ability to climb. Scoring based on counting the number of flies that stayed at the bottom of the tube demonstrated that this approach could be used to measure sensitivity to a single exposure. This approach proved adequate for a genetic screen to identify flies with changed sensitivity, and resulted in the identification of several candidate genes that were functionally conserved in rodents (Tsai et al., 2004; Bainton et al., 2005; Schwabe et al., 2005).

George et al. (2005) implemented a semi-automated procedure for bottom counting of affected flies and showed that such quantification can be used to measure behavioral sensitization after multiple exposures. Furthermore, this method has specificity to identify a circadian mutant *per^*0*^*, with the same sensitivity as the previous method that relied on visual scoring of the affected behavior (Andretic et al., 1999). However, the semi-automated bottom-counting method has never been used in a genetic screen. Potential reasons are that setting-up the bottom-counting method requires engineering expertise, the method does not improve on variability that arises from potential uneven cocaine amount that each fly receives, and quantification is population based.

Ultimately, methodological improvements in drug delivery and quantitative measurement of the consequences of drug delivery were never successfully integrated into a single high-throughput method. Other improvements were made that relied on video tracking of cocaine-induced behavior, but these methods required significant animal handling and were too time consuming for a genetic screen (Bainton et al., 2000; Gakamsky et al., 2013). Consequently, identification of novel genes involved in drug-induced neural plasticity has stalled due to the lack of an adequate behavioral test for use in a genetic screen.

Here, we present a new method of inducing locomotor sensitization to volatilized cocaine (vCOC) in flies, named FlyBong. This technique delivers a precise amount of volatilized drug to each fly, quantifies in a dose-dependent manner the locomotor activity of individual fly, and compares the change in locomotor activity of individual fly before and after the administration. Because of its high-throughput nature, it can be used in genetic screens to monitor changes in locomotor activity to single or multiple exposures of different psychoactive chemicals with motor-activating effects.

## Materials and Methods

### Fly Strains

All behavior experiments were performed on 3- to 5-day-old wild type (wt) flies with a CantonS background. Flies were raised on a standard cornmeal/agar medium in LD 12:12 at 25°C, 70% humidity. Circadian mutant strains used were: *per^*01*^*, *tim^*01*^*, *cyc^*01*^*, *Clk^Jrk^* and *pdf^*01*^* generously donated by C. Helfrich-Forster and mutation in dopamine transporter, *fumin* (*fmn*) from S. Birman [21]. Flies were collected using CO2 anesthesia 1 day before the experiment, and following day were transferred using an aspirator into Drosophila Activity Monitor (DAM) system tubes.

### Chemicals

Cocaine-hydrochloride was purchased from Sigma–Aldrich (≥97.5%) and 96% ethanol used for preparing 10 mg/mL stock solution was purchased from VWR.

### FlyBong Procedure

The FlyBong platform involves volatilizing a specific concentration of cocaine in a three-neck glass flask and then pumping the aerosol for 1 min into a collection of polycarbonate tubes, each housing single flies in a vertical DAM. Locomotor activity is measured as the number of times that an individual fly crosses the midline of the tube per minute. When quantifying the effect of volatilized drug on the behavior of flies, the average level of locomotor activity after exposure is compared to that before drug exposure (the baseline), while specificity of the cocaine effect is determined by comparison to the locomotor activity of a population exposed to warm air without cocaine (the control). Population response involves comparing average locomotor activity of varying length of time post-exposure (up to 30 min) to the average pre-exposure, baseline or control levels.

**FIGURE 1:**
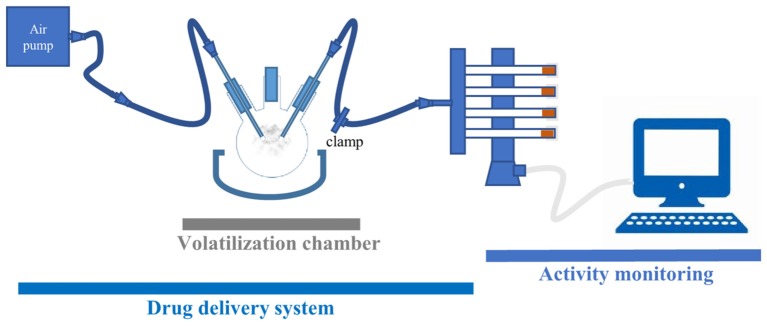
FlyBong platform for measuring changes in locomotor activity of *Drosophila* after delivery of vCOC. Cocaine dissolved in ethanol is pipetted into three-neck flask, which is then heated to the cocaine volatilization temperature. Single flies are housed in the individual tubes of the vertical *Drosophila* monitor (TriKinetics) and exposed to vCOC for 1 min by turning on the air pump and removing the clamp. Their locomotor activity is monitored as the number of crossings of the midline of the tube per minute for 30 min before and 30 min after vCOC exposure.

The platform for psychostimulant administration and locomotor activity monitoring consists of two main components: a drug delivery and a locomotor activity monitoring part (**Figure 1**). The drug delivery part consists of volatilization chamber (a three-neck flask, 250 mL, VWR), connected on one side to an air pump (Crawfish 1800 air pump), and on the other to a Gas Distribution Manifold (TriKinetics). The locomotor monitoring part consists of a manifold that delivers air to polycarbonate tubes (with two small holes to allow airflow), housing single flies in a vertical DAM (all from TriKinetics). Every DAM system tube has food on one end to prevent starvation or dehydration of flies during the assay. Locomotor activity counts are collected every minute on a computer using a PSIU9 Power Supply Interface Unit (TriKinetics). The connection between the volatilization chamber and DAM is clamped, except during the exposure of flies to a mild stream of air or vCOC.

Cocaine is volatilized in a three-neck flask positioned in a heat cap (SAF, LabHEAT, KM-G, for 250 mL flask). The central neck of the flask is used for pipetting in the cocaine–ethanol solution. To eliminate potential effects of ethanol, cocaine dissolved in ethanol is added 4–6 h before drug administration to allow the ethanol to vaporize. The flask is then closed and heated for 8 min, at which point it reaches 185°C, while the tube leading to the DAM monitor is clamped. After 8 min heating, the cap is turned off, the clamp removed and the aerosol is pushed into DAM for 1 min using an air pump with a flow rate of 2.5 L/min.

Multiple administrations of COC were performed using the same standard protocol as single administrations (1 min, 2.5 L/min), but with lower COC concentration (45 μg). COC was administered three times: first at 9:00 AM, second at 7:00 PM and third 9:00 AM of the following day.

To test for circadian modulation of sensitivity and locomotor sensitization, we performed the assay in constant dark conditions (DD), using the standard protocol and with 12 h between exposures. Flies previously entrained to 12 h light and 12 h dark (LD) were then released in constant dark (DD) for 1 day before the experiment. The first group of flies in DD was exposed to the first dose of COC at 10 am (2 h after light would have come on in LD) and to the second at 10 pm (2 h after light would have come on in LD). The second group received the first dose at 10 pm and the second at 10 am. All experimental manipulations were performed under dim red light.

### Cocaine Measurements

To test the homogeneity of distribution and stability of COC after volatilization, as well as the amount delivered to the individual flies we measured amount of COC in the tube without the fly, and separately the amount of COC on the fly. COC amount inside the tube was determined by using empty tubes without flies or food, connected to the FlyBong on one side and immersed in 2 mL Eppendorf tubes filled with 500 μL of distilled water on the other side. We applied standard protocol (8 min of volatilization, 2.5 L/min air flow, 1 min of drug delivery, and 75 μg of COC). After 5 min, all tubes were detached from the dispenser and washed with an additional 500 μL of distilled water. Eppendorf tubes with volatilized samples were additionally vortexed for 1 min at 2,500 rpm. The monitor and dispenser are arranged with four rows and eight columns in each row, so that for all 32 samples the UV-VIS spectra was recorded in triplicate. Five hundred microliters of each sample was recorded using a 48-well plate and microplate reader TecanInfinitePro200 at λ_max_ = 275 nm, is the maximum absorbance for COC in UV-VIS spectra.

To measure COC amount on the surface of the fly we applied standard protocol to 32 flies housed in individual tubes of DAM monitor. Five min after the exposure each fly was frozen at -20°C. Each fly was then transferred to the 2 mL Eppendorf tube filled with 300 μL of distilled water. Tubes containing flies were vortexed for 1 min at 2,500 rpm. Two hundred and fifty microliters of each sample was recorded in triplicate using a 48-well plate and microplate reader TecanInfinitePro200 at λ_max_ = 275 nm.

Concentration of COC in the samples (on the flies or inside the tube) was determined using COC calibration curve. Calibration curve was determined from standards with serial dilution of COC concentration by measuring the absorbance at 275 nm. Using the linear regression method we derived the equation for determining COC concentration in the samples. To test the stability of COC after volatilization, we performed UV-VIS on random triplicate of COC samples after volatilization using Cary 60 UV-VIS in range from 200 to 800 nm and compared it to COC standard made from COC that has not undergone 8 min heating procedure.

### Data Analysis

The DAM system collects data as a number of times that an individual fly crosses the midline of its tube in 1 min, which allows the data to be analyzed at either a population or an individual fly level. For analysis of population data, data was collected for 30 min before and 30 min after drug delivery (heating and drug delivery time are excluded from this analysis), allowing calculation of average counts per minute for 32 flies. Data can then be plotted as an average of counts per minute for 32 flies in different time intervals before and after administration.

Individual fly response compares average counts per minute of an individual fly in the first 5 min before (baseline) and after cocaine administration (excluding heating and drug delivery time). The baseline locomotor activity is then compared to activity after administration and the activity is qualified as either: the same, decreased, increased or other (flies with inconsistent changes after subsequent administrations). The number or percentage of flies with an increased activity after first drug exposure, compared to their baseline, represents a measure of sensitivity (SENS) to cocaine. After the second drug administration, locomotor activity after administration is compared to locomotor activity after first exposure. The number or percentage of flies that develop locomotor sensation (LS), is based on whether each individual fly displays increased locomotor activity after first administration, relative to baseline, and a further increase after the second administration, relative to the first. These same criteria were used for calculating locomotor sensitization of individual flies after multiple doses of psychostimulant.

### Statistical Analyses

Statistical analyses of population data were performed using Student’s *t*-test for independent (between groups) or dependent (within same group) samples. Individual data was statistically tested using χ^2^ test. Differences were considered significant if probability of error was ≤0.05.

## Results

### Protocol Optimization

To develop the standard protocol, which consists of heating the flask for 8 min, followed by cocaine delivery for 1 min using 2.5 L/min airflow, we performed a series of control experiments. These experiments showed that changes in locomotor activation following cocaine administration are specific to the activating effects of cocaine and not dependent on other environmental factors, as detailed below.

**FIGURE 2:**
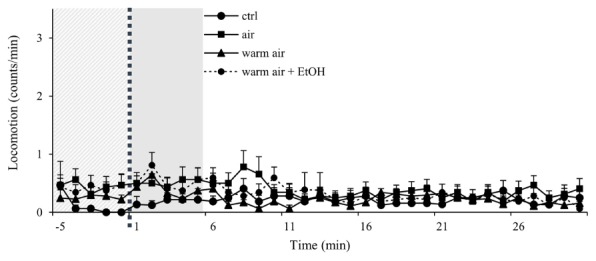
Control experiments show minimal disturbance of locomotor activity in *wt* males. Kinetic line graphs of locomotor activity in different control groups. Group no treatment represent flies with normal activity without any experimental exposures, air - 2.5 L/min air flow for 1 min, warm air - 2.5 L/min air flow for 1 min, following 8 min of heating, warm air + EtOH - 2.5 L/min air flow for 1 min following 8 min of heating of 75 μL of 96% ethanol. Experiments *n* = 32 flies per group, were repeated for each group three times. Light gray panel represents locomotor activity 5 min before exposure for all groups, dotted line is time of the exposure, and darker gray panel denotes 5 min after exposure. Data are plotted as mean + SEM of counts/min, with a resolution of 1 min.

Firstly, we compared the locomotor activity of undisturbed group to groups that received either air pumped in without heating of the flask, heated air as in the standard protocol or heated air with un-vaporized ethanol. Ethanol is used to dissolve cocaine and to deliver it to the flask, however in all experiments, we wait for the ethanol to vaporize before heating the flask. Neither of these conditions led to a significant change in the baseline activity (**Figure 2**).

Cocaine hydrochloride starts to volatilize at around 185°C. For our protocol, we discovered that the three-neck flask, has to be heated for 8 min in order for the internal temperature to reach 185°C (Supplementary Figure 1A). At this time point the flasks fills with a visible cloud of cocaine aerosol.

To test if the distribution of cocaine was homogenous between each tube of the recording monitor we measured the amount of cocaine residues in each tube and on each fly (see Materials and Methods), and determined using calibration curve (Supplementary Figure 1B) that the concentration of cocaine in the tubes did not depend on tube location and that each fly received to the same amount of vCOC (Supplementary Figure 1C). Thus, the individual differences in the amount of locomotion that vCOC induces cannot be explained with the variability of the amount of delivered cocaine. To confirm that volatilization procedure did not caused decomposition of COC we compared UV VIS spectra of cocaine samples from the experiment, to cocaine standard and determined that only peaks at 230 and 275 nm were present in all spectra (Supplementary Figure 1D).

We tested several intensities of airflow without cocaine or heating, in order to determine potential effect of airflow on locomotor activity. The maximal airflow that could be used without causing an increase in the locomotor activity of the flies was 2.5 L/min (Supplementary Figure 2). To determine the optimal duration of airflow, we heated the flask without cocaine for 8 min, activated the air pump at a flow rate of 2.5 L/min and exposed flies to warm airflow for various time periods of between 10 s and 7 min (Supplementary Figure 3A). One-minute long exposure time led to the least disturbance in locomotor activity of the flies, with no significant difference from baseline activity in 5 min before exposure (Supplementary Figure 3B). In contrast, durations shorter or longer than 1 min led to a significant increase in locomotor activity (Supplementary Figure 3B). We suspect that shorter exposures (<1 min) cause a startle-like response, while longer exposures (>1 min) decreased humidity and increased temperature in the recording tubes, causing increased locomotion.

### Acute Administration of vCOC Increases Locomotor Activity

**FIGURE 3:**
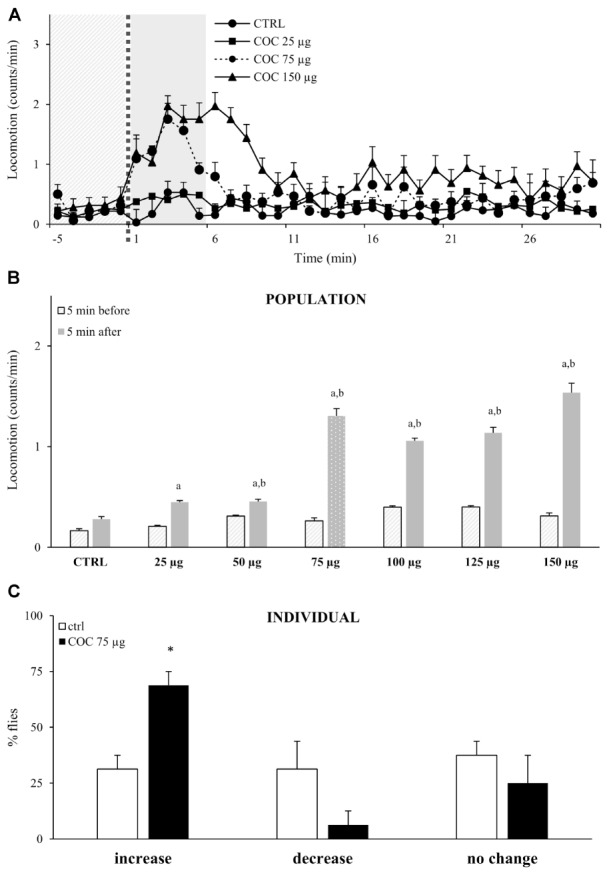
Exposure to vCOC transiently and dose-dependently increases locomotor activity. **(A)** Kinetic graph of locomotion expressed as number of counts per minute for a control group of flies exposed to warm air (ctrl) and of groups exposed to vCOC concentrations of 25 μg (COC 25 μg), 75 μg (COC 75μg), or 150 μg (COC 150μg). Experiments *n* = 32 flies per group, were repeated for each group three times The light gray panel indicates the 5 min immediately prior to exposure, the dotted line is the time of exposure and the dark gray panel indicates 5 min after exposure. **(B)** Mean locomotor activity + SEM for vCOC concentrations ranging from 25 to 150 μg (*n* = 32 flies per treatment) for the 5 min immediately before and after cocaine exposure. Statistical significance (*p* ≤ 0.05) indicated by a: within group comparison of locomotion before and after administration (*t*-test for dependent samples); b: between group comparison for 5 min after exposure (*t*-test for independent samples). **(C)** Changes in level of locomotor response of individual flies where amount of locomotor activity for individual fly after exposure to 75 μg of vCOC (8 min heating, 1 min exposure and 2.5 L/min air flow) or warm air control (ctrl) (*n* = 32 for each group). Changes are categorized as increase, decrease or no change, based on locomotion in the 5 min immediately after exposure, compared to the 5 min before. ^∗^χ^2^ test statistical significance (*p* ≤ 0.05) between group comparison.

To test if exposure to vCOC has dose-dependent motor-activating effects, we exposed flies to vCOC concentrations ranging from 25 to 150 μg using FlyBong. Flies that were exposed to cocaine displayed increased locomotor activity in the first minute after cocaine delivery had ceased, and locomotion remained significantly elevated for 5 min (**Figure 3A**). This increase is statistically significant when compared to the pre-exposure baseline level and levels in the control group, Student’s *t*-test for dependent samples *p* < 0.05 (**Figure 3B**). After 5 min, levels of locomotor activity were once again similar to pre-exposure baseline and to the post-exposure levels in the control group, indicating a transient effect of acute cocaine administration on locomotion.

Although flies responded with an increased amount of locomotion to the lowest dose (25 μg) of vCOC, we observed a robust response to 75 μg vCOC, while higher doses did not lead to further significant increases in locomotion (**Figure 3B**). The absence of a wider range of linear dose-dependent increases in locomotor activity likely reflects the appearance of motor behaviors, besides locomotion. Depending on the dose, vCOC can induce a range of hyperactive and stereotypical behaviors (McClung and Hirsh, 1998), that would not lead to linear increases in locomotion as measured by DAM monitors.

We next sought to determine the extent to which individual responses correspond or contributed to the population average, by analyzing locomotor activities of individual fly during the 5 min interval immediately before (baseline) or after exposure to 75 μg of vCOC. We categorized each locomotor response as either an increase, decrease, or no change when compared to baseline levels. The majority of individual flies respond with an increased locomotion after cocaine exposure (69%), and only a few (6%) with a decrease, a statistically significant difference *p* < 0.05 χ^2^ test, while the remaining showed no change in level of locomotion (25%) (**Figure 3C**). Flies with an increased response represent individual sensitivity to motor-activating effects of cocaine, and the percentage of flies with such as response in this experiment demonstrates that cocaine had a motor activating influence in the majority of flies. In the control group, which was exposed to warm air only, the percentage of flies that increased, decreased or did not change their locomotion was approximately equal, suggesting a random effect (**Figure 3C**).

Female flies showed distinct locomotor responses to acute vCOC when compared to males. Similar to males, their locomotor activity is transiently increased immediately after exposure, and shows a dose-dependent increase in locomotion when exposed to 75 and 100 μg of cocaine (Supplementary Figures 4A,B). However, female flies showed altered locomotor activity following 1 min exposure to warm airflow alone, in contrast to the males, and it is therefore not clear what proportion of their response is cocaine specific (Supplementary Figures 5A,B and 4B). Furthermore, analysis of the responses of individual flies showed that their sensitivity to warm airflow alone is similar to their sensitivity to vCOC (Supplementary Figures 4B,C). These data demonstrate a pronounced sexual dimorphism in the motor-activating effects of cocaine in *Drosophila*, as reported previously (McClung and Hirsh, 1998). For that reason all currently published reports on the behavioral effect of vCOC are done using only male flies. Since in our experiments males also show a reliable and cocaine-specific increase in locomotion after exposure, all further experiments were performed using male flies only.

### Repeated Administration of vCOC Leads to Locomotor Sensitization

**FIGURE 4:**
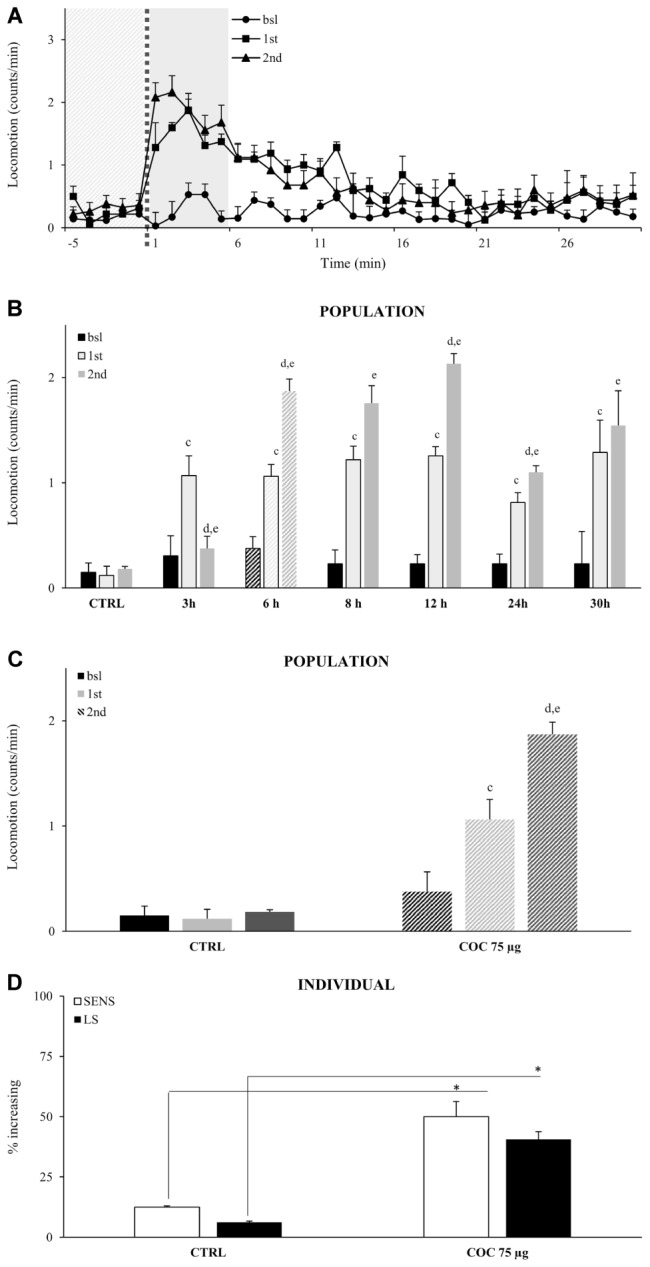
Locomotor sensitization to vCOC depends on time interval between exposures wile only limited number of individual flies in a population develops locomotor sensitization to vCOC. **(A)** Kinetic graph of average locomotion (counts per minute) for group of flies (*n* = 32) before administration (bsl), and after administrations of 75 μg vCOC first at 09:00 AM (1st) and then a second administration at 03:00 PM (2nd). Light gray panel 5 min before exposure, dotted line is time of the exposure, dark gray panel 5 min after exposure. **(B)** Histogram of different duration of time intervals between two administrations of vCOC (75 μg), plotted as a mean of population (32 flies) locomotor activity 5 min before (bsl) and 5 min after COC (1st) exposures at 09:00 AM and 2nd at different time points [12:00 AM (3 h), 03:00 PM (6 h), 05:00 PM (8 h), 09:00 PM (12 h), 09:00 AM of the following day (24 h) and 03:00 PM of the following day (30 h)], +SEM. The control (CTRL) group was exposed to warm air (8 min of heating, 1 min exposure, and 2.5 L/min air flow rate). Statistical significance (*p* ≤ 0.05) indicated by: c: within group comparison of bsl with 1st; d: 1st with 2nd; e: bsl with 2nd (*t*-tests for dependent samples). **(C)** Population response of the control group (CTRL) exposed to warm air and a group exposed twice to vCOC (COC 75 μg) using our standard protocol, with the first administration at 9:00 AM and the second at 3:00 PM. Data are plotted as mean of population (32 flies) locomotor activity 5 min before and 5 min after (1st and 2nd) exposure to COC, +SEM. Statistical significance (*p* ≤ 0.05) indicated by: c: comparison of bsl with 1st; d: 1st with 2nd; e: bsl with 2nd (within group comparison using *t*-test for dependent samples). **(D)** Analysis of individual flies from data in part **(A)**. Percent of individual flies in a population that show sensitivity (SENS) and locomotor sensitization (LS) in control (CTRL) and group exposed to vCOC (COC 75 μg). ^∗^χ^2^ test statistical significance *p* ≤ 0.05. Starting data were same as **(C)** from which categories increase, decrease and same were divided.

Repeated administrations of the same concentration of vCOC leads to locomotor sensitization, as demonstrated by an increase in the percentage of flies exhibiting hyperactive and uncoordinated behaviors (McClung and Hirsh, 1998). To test if we can induce and measure locomotor sensitization using FlyBong, we exposed *wt* male flies to two doses of vCOC using the standard protocol (75 μg of cocaine, 8 min of heating, 1 min exposure and 2.5 L/min air flow), but using variable time intervals between the exposures, ranging from 3 to 30 h. The first administration was always at 9:00 AM, 1 h after the lights were switched on. A significant increase in the amount of locomotor activity in response to the second dose, when compared to the first, was present when administrations were given between 6 and 24 h apart, Student’s *t*-test for dependent samples *p* < 0.05 (**Figures 4A,B**), indicating that flies develop locomotor sensitization to vCOC. Locomotor sensitization is a long-term change in the responsiveness of the nervous system, and its presence in these flies is supported by the observation that the earliest time point at which significant locomotor sensitization occurs is 6 h after the first exposure. At earlier time points, we observed a decrease in locomotor activity, suggesting that the time interval was insufficient for expression of locomotor sensitization.

Locomotor sensitization can emerge from the dynamics of locomotor responses of a population (**Figure 4C**), however, it is not known to what extent the behavioral sensitization of individual flies contributes to the population response. We define locomotor sensitization of an individual fly as an increase in locomotor activity after the first administration (compared to baseline) combined with a further increase following the second administration (compared to the first). According to these criteria, 40% of the flies showed locomotor sensitization to vCOC (**Figure 4D**) compared to controls where only 6% of flies showed locomotor sensitization, a statistically significant difference *p* < 0.05, χ^2^ test. Thus, while sensitivity (increased locomotor responding to a single dose) is a prevalent response in a population of the flies (69% flies), only a subgroup of those flies (40%), could be seen to develop locomotor sensitization (**Figure 4D**).

We applied the same standard protocol to *wt* females but did not observe the appearance of locomotor sensitization. At the population level, the second administration of cocaine did not induce an increase in locomotor activity, and analysis of individual flies shows that the experimental group did not differ significantly from the control (Supplementary Figures 6A,B). These results do not necessarily imply that locomotor sensitization cannot be induced in females, but rather that our standard protocol is not optimized for females.

**FIGURE 5:**
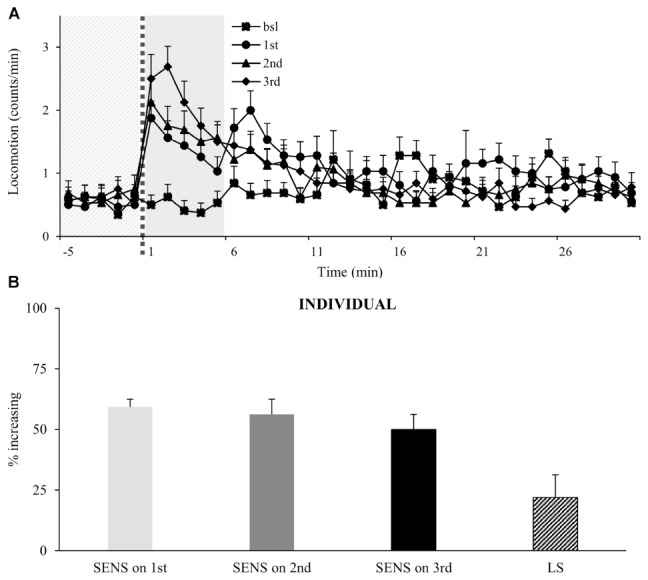
Multiple exposures to cocaine increases locomotor activity of a population, while sensitivity of individual flies does not change. **(A)** Kinetic graph of mean locomotion (counts/minute) for group of flies (*n* = 32) both before (bsl) administration, and after exposure to volatilized 45 μg cocaine on three occasions: the first at 09:00 AM (1st), the second at 07:00 PM (2nd) and the third at 09:00 AM of the next day (3rd). The light gray panel indicates the 5 min immediately prior to exposure, the dotted line is the time of exposure and the dark gray panel indicates 5 min after exposure. **(B)** Analysis of individual flies from data in part **(A)**. Percentage of individual flies in population which show sensitivity (SENS) and locomotor sensitization (LS). Starting data were same as **(A)** from which categories increase, decrease and same were divided.

To test if flies will continue to increase their locomotor activity following multiple administrations, we exposed flies to three intermittent doses of vCOC. Analysis of the population shows that flies continue to increase their locomotor activity following the third exposure (**Figure 5A**), however, an important characteristic of individual response is not obvious from the population data. Specifically, the percentage of flies that are sensitive to each given administration is similar for all three doses and varies by around 50%, but of these, only a subpopulation (22%) showed the consecutively increased locomotion to each dose that would indicate locomotor sensitization in this assay (**Figure 5B**).

### Locomotor Sensitization to vCOC Depends on Functional Circadian Genes and Dopaminergic Transporter

Studies in both *Drosophila* and laboratory rodents have shown that the genes that control circadian rhythmicity are involved in the regulation of motor activating and in arousing effects of psychostimulants (Andretic et al., 1999, 2005). To validate FlyBong and to measure locomotor sensitization we exposed light:dark entrained flies with mutantions for circadian genes *per^*01*^*, *tim^*01*^*, *Clk^Jrk^* and *cyc^*01*^* to vCOC. We also include flies with a mutation in a gene for a neuropeptide pigment dispersing factor (*pdf*), that conveys signals from circadian pacemaker cells in the fly brain to the rest of the brain and body (Renn et al., 1999). PDF-positive neurons also express *dLmo* gene, a regulator of LIM-homeodomain proteins, identified as a regulator of cocaine sensitivity in *Drosophila* (Tsai et al., 2004).

**FIGURE 6:**
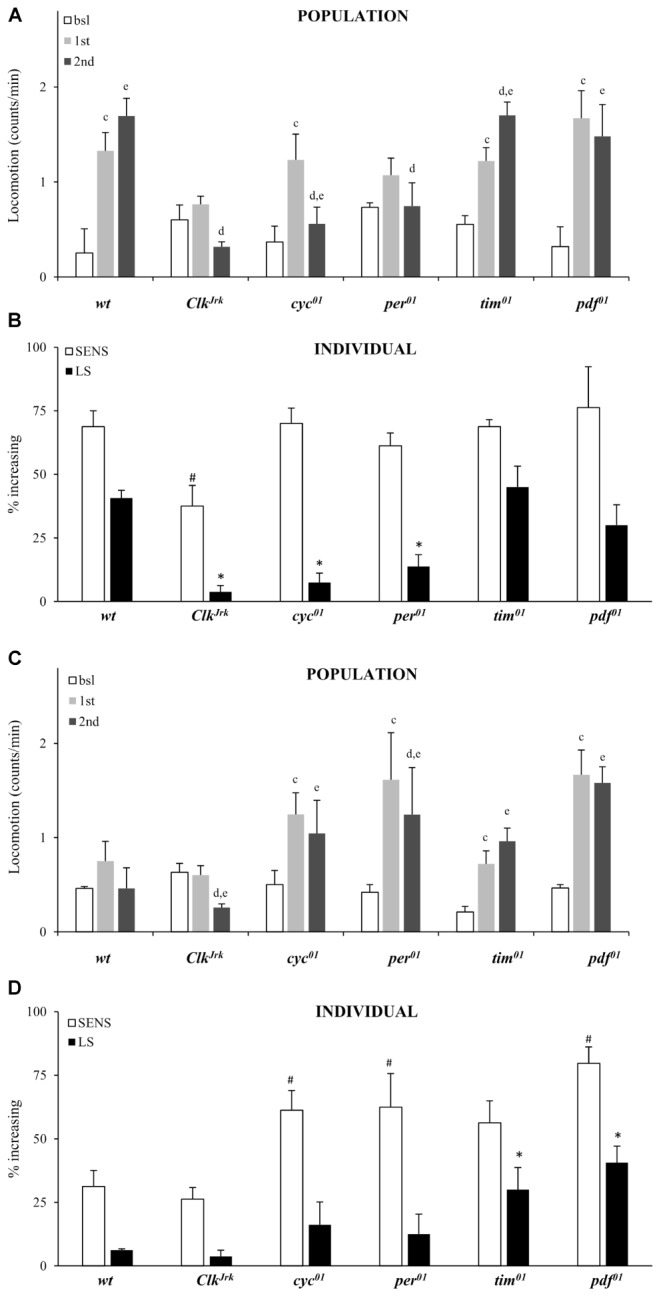
Locomotor sensitization to vCOC depends on circadian genes. **(A)** Average locomotor activity of flies 5 min before drug exposures (bsl), 5 min after first exposure (first) and 5 min after second (second) exposure to 75 μg of vCOC given 6 h apart. Fly populations were either *wild type* (*wt*) or mutant for circadian genes *Clk^Jrk^*, *per^*01*^*, *cyc^*01*^*, *tim^*01*^* and *pdf^*01*^* (*n* = 32 for each group). Statistical significance (*p* ≤ 0.05) indicated by: c: comparison of baseline to after first administration; d: after first to after second and e: baseline to after second administration (all within the group using *t*-test for dependent samples). **(B)** Percentage of individual flies showing sensitivity or increasing locomotor activity to the first exposure of vCOC (bsl vs. 1st) and flies showing further increase in locomotor activity to the second exposure (LS) (bsl vs. 1st vs. 2nd). χ^2^ test showed statistical significance *p* ≤ 0.05 for comparison of wt to mutants in SENS (#) and (^∗^) LS. Starting data were same as **(A)** from which categories increase, decrease and same were divided. **(C)** Average locomotor activity (counts/min) during baseline, 5 min before exposures (bsl), 5 min after first exposure (1st) and 5 min after second (2nd) exposure to warm air flow (2.5 L/min, for 1 min after 8 min of heating), given 6 h apart. Fly populations were either *wild type* (*wt*) or mutants for circadian genes *Clk^Jrk^*, *per^*01*^*, *cyc^*01*^*, *tim^*01*^*, and *pdf^*01*^* (*n* = 32 for each group). Statistical significance (*p* ≤ 0.05) indicated by: c: comparison of baseline activity to after first administration; d: comparison of activity after first and second exposures; e: comparison of baseline activity to after second administration. All tests were within the group, using *t*-test for dependent samples. **(D)** Percentage of individual flies showing sensitivity or increased locomotor activity to a first exposure to warm air flow (2.5 L/min, for 1 min after 8 min of heating) and flies showing further increase in locomotor activity to a second exposure (LS). χ^2^ test showed statistical significance (*p* ≤ 0.05) for comparison of wt to mutants in SENS (#) and (^∗^) LS. Starting data were same as **(C)** from which categories increase, decrease and same were divided.

At the population level we confirm that the *per*, *Clk* and *cyc* genes are required for locomotor sensitization to vCOC. Exposure to the standard protocol with two doses of vCOC 6 h apart (first administration at 9:00 AM, second at 3:00 PM) did not lead to increased locomotor activity to successive doses of vCOC in *per*^*01*^, *Clk^Jrk^* and *cyc^*01*^* mutants when locomotor activity after first exposure was comparied to locomotor activity after second exposure using *t*-test for dependent samples (**Figure 6A**). We also confirm that *tim^*01*^* males behave similar to *wt*, suggesting that *tim* gene is not required for development of behavioral sensitization (**Figure 6A**). At the population level we also observe lack of locomotor sensitization in *per*^*01*^ mutant males.

Consistency between individual and population level analysis depended on the mutant strain (**Figures 6A,B**). Individual analysis confirmed the importance for *per*, *Clk* and *cyc* genes and showed that the lack of locomotor sensitization in *Clk^Jrk^* flies is accompanied by a decreased sensitivity to the first administration of vCOC determined using χ^2^ test (**Figure 6B**). However, there was a discrepancy between population and individual level analysis of *pdf^0^* mutants. While *per*^*01*^ flies did not develop locomotor sensitization at the population level, analysis of individual flies showed the contrary (**Figures 6A,B**).

To determine the origin of this discrepancy, we analyzed population and individual response of all flies to two exposures of warm air. The *Clk^Jrk^* mutant was similar to *wt* flies and is not sensitive to, nor develops locomotor sensitization to, the warm air (**Figures 6C,D**). Over 50% of the *per^*01*^*, *cyc^*01*^*, *tim^*01*^* and *pdf^*01*^* populations showed sensitivity to warm air, while over 30% of *tim^*01*^* and *pdf^*01*^* mutant flies develop locomotor sensitization to warm air (**Figure 6D**). These data suggest that the putative locomotor sensitization of *tim^*01*^* and *pdf^*01*^* mutants to cocaine has to be reexamined, as at least part of that phenotype is not cocaine-specific. Especially in regard to *pdf^*01*^* mutants, where population and individual results diverged, there is the need for further examination of the interaction between the output from the central circadian pacemaker and the regulation of locomotor sensitization to vCOC. Very low fraction of *per^*01*^*, *Clk^Jrk^* and *cyc^*01*^* flies develops locomotor sensitization to vCOC as does to warm air (**Figure 6**), arguing for impaired mechanisms of neuronal plasticity in these mutants. Individual level analysis of FlyBong locomotor output and use of appropriate controls offers new information about behavioral responses, that would not be obvious when examining only the average population response to vCOC.

Our data suggests that the ability to develop locomotor sensitization depends on the presence of functional circadian genes, possibly regulating circadian behavior separately from drug-induced behavior. However, this does not exclude a rhythmic circadian modulation of sensitivity to an acute dose of cocaine, or neuronal plasticity after multiple doses. To test if sensitivity or locomotor sensitization is influenced by circadian modulation, we administered two doses of vCOC with a 12-h interval. These experiments were performed in constant darkness to remove the confounding factor of light and to demonstrate if the source of modulation is the activity of the endogenous circadian clock.

**FIGURE 7:**
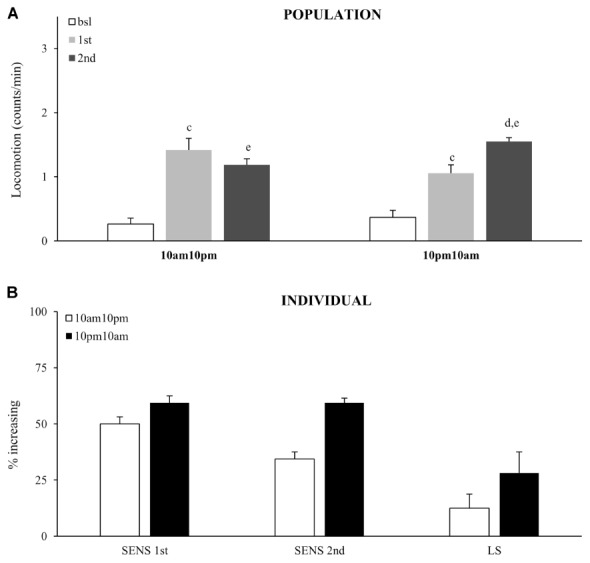
Time of day modulates locomotor sensitization, without affecting sensitivity to the first exposure. **(A)** Average locomotor activity (counts/min) during baseline, 5 min before drug exposures (bsl), 5 min after first exposure (1st) and 5 min after second (2nd) exposure to 75 μg of vCOC given 12 h apart for of a population of wild type (wt) (*n* = 32 for each group). Statistical significance (*p* ≤ 0.05) indicated by: c: comparison of baseline to after first administration; d: after first to after second; e: baseline to after second administration (all within the group using *t*-test for dependent samples). **(B)** Percentage of individual flies showing increase in locomotor activity to the first exposure of vCOC compared to baseline (SENS 1st), percentage of flies that show increase in locomotor activity to the second exposure, relative to the first (SENS 2nd), and a subgroup that shows increase after first and further increase after second exposure (LS). Starting data were same as **(A)** from which categories increase, decrease and same were divided.

Sensitivity to the acute dose did not vary as a function of the circadian time, as the percentage of flies that responded with increased activity at 10:00 AM did not vary significantly from 10:00 PM, χ^2^ test (**Figures 7A,B**). The same result was obtained from both population and individual level analysis. Interestingly, fewer flies showed an increased response to the second dose (relative to the first) when it was given at 10:00 PM, which consequently resulted in a weaker locomotor sensitization. Thus, expression of locomotor sensitization is weaker during the subjective night, than during the subjective day. This shows that circadian clock modulates cocaine induced neuronal plasticity, but not sensitivity to cocaine.

**FIGURE 8:**
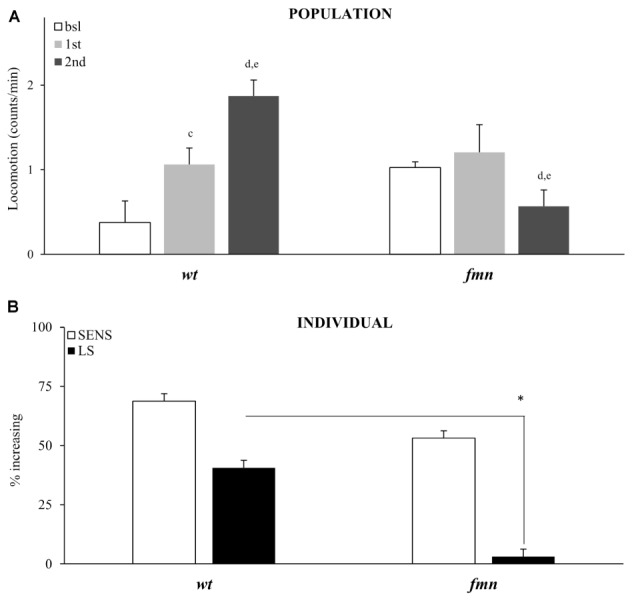
Locomotor sensitization to vCOC is dependent on a functional dopaminergic transporter. **(A)** Average locomotor activity (counts/min) during baseline, 5 min before drug exposures (bsl), 5 min after first exposure (1st) and 5 min after 2nd) exposure to 75 μg of vCOC given 6 h apart for a population of wild type (wt), and flies mutant in dopaminergic transporter, *fumin* (*fmn*) (*n* = 32 for each group). Statistical significance *p* ≤ 0.05; c: comparison of baseline to after first administration, d: after first to after second and e: baseline to after second administration (all within the group using *t*-test for dependent samples). **(B)** Percent of individual flies increasing locomotor activity to first exposure of vCOC (bsl vs. 1st) and flies showing further increase in locomotor activity to the second exposure (LS) (bsl vs. 1st vs. 2nd). χ^2^ test showed statistical significance *p* ≤ 0.05 (^∗^) for wt compared to *fmn* in LS. Starting data were same as **(A)** from which categories increase, decrease and same were divided.

Sensitivity to the motor-activating effects of cocaine in mammals depends on a functional dopaminergic transporter (DAT), a direct molecular target for cocaine (Chen et al., 1992; Giros et al., 1996; Mead et al., 2002; Makos et al., 2010). *Drosophila* with a mutation in a DAT gene named *fumin* (*fmn*), are hyperactive and have reduced sleep due to a decreased dopamine uptake into the presynaptic cell (Kume et al., 2005; Makos et al., 2010; Ueno and Kume, 2014). To further validate our platform and to show that motor-activating effects of vCOC that we measure depend on functional DAT we exposed *fmn* flies to two doses of vCOC, 6 h apart. Population level analysis showed that *fmn* flies indeed have increased basal levels of activity when compared to *wt* controls, and are not sensitive to acute exposure to 75 μg of vCOC, Student’s *t*-test for dependent samples (**Figure 8A**). A second cocaine exposure leads to a decrease in the average amount of locomotor activity. These data suggest that a functional DAT protein is required both for sensitivity to cocaine and for development of locomotor sensitization (**Figure 8A**). However, individual level analysis showed that the flies sensitivity to the first cocaine administration is preserved (**Figure 8B**). As above for the *pdf* mutant, this can be explained by increased sensitivity to the warm air (Supplementary Figures 7A,B), which is in accordance with the heightened arousal of these flies.

## Discussion

### FlyBong Is Suitable for Genetic Screens and Selection Experiments

We have developed a new method for quantifying vCOC-induced changes in the locomotor activity of *Drosophila*, that we name FlyBong. The method consists of a novel drug delivery system, connected to a commercially available platform for quantifying the locomotion (TriKinetics). Using FlyBong we can, with minimal animal handling: (a) deliver precise drug amount to individual flies, (b) objectively quantify the level of locomotor activity both before and after drug administration, (c) quantify the change in locomotor activity of either single flies or the whole population, (d) record locomotor activity of 32 flies simultaneously. With only a moderate financial investment, FlyBong could easily be scalable up, allowing it to be used in genetic screens in which hundreds of flies could be tested per day. These characteristics make FlyBong significantly more objective, efficient and versatile, as well as higher throughput, than previously described methods.

The most important improvement of FlyBong, relative to previous systems, is that it provides both population and individual measures of locomotor activity. We have shown that there is individual variability in flies’ sensitivity to motor-activating effects of cocaine, and that only a fraction of these sensitive flies go on to develop locomotor sensitization. This suggests that neural mechanisms that govern sensitivity are distinct from those that regulate locomotor sensitization. Several results described here support this conclusion: First, three consecutive exposures to vCOC each led to similar percentage of flies showing sensitivity, while the percentage of flies that develop locomotor sensitization to the three exposures is much lower. Second, sensitivity does not vary as a function of circadian time, while locomotor sensitization does. Third, although sensitivity of *per* and *cyc* mutant flies is similar to that of *wt* flies, they fail to develop locomotor sensitization.

The reason that vCOC was initially selected as a drug of choice for *Drosophila* was that it led to dramatic and concentration-dependent changes in motor behavior, as exemplified by the appearance of uncoordinated, uncommon or stereotypical behaviors (McClung and Hirsh, 1998). Increases in forward locomotion were observed at lower doses, and has been quantified using methods based on oral administration of cocaine or its injection into the abdomen (Dimitrijevic et al., 2004; Lebestky et al., 2009). Transition from increased locomotion into uncoordinated or stereotypical behavior is dose-dependent. As we have used a DAM monitor to quantifying locomotor activity, we are aware of these limitations and have identified cocaine concentrations that lead to dose-dependent change in locomotor activity, but not in other uncoordinated behaviors. We have unpublished evidence that other psychoactive compounds with a predominant locomotor effect, lead to more significant dose-dependent and sensitizing effects.

The 75 μg does of vCOC used in our experiments is the same as has been used in other experiments in which it was delivered from a heated filament positioned in close proximity to a group of 10 flies in a vial (McClung and Hirsh, 1998, 1999; Andretic et al., 1999; Li et al., 2000; Park et al., 2000). In FlyBong, cocaine is volatilized in a flask and delivered via tubing and a dispenser to individual flies in recording tubes, although a proportion of vCOC instead condenses on the surfaces through which it flows before it reaches the recording tubes. We have therefore measured residual cocaine on the surface of the recording tubes and showed that less than 10% of the volatilized amount reaches the individual tube. This quantity is within the range of concentrations that predominately induces locomotor activation. Absence of a wider ranging dose-response curve using FlyBong argues for the appearance of uncoordinated behaviors at higher doses, which in our system cannot be recorded as an increase in locomotion.

Delivering a precise drug amount to individual flies within a population had not been achieved using previously published methods (McClung and Hirsh, 1998; Bainton et al., 2000, 2005; George et al., 2005). Attempts to deliver a known amount of drug via injection or an air brush were not simple and required sophisticated animal handling (Dimitrijevic et al., 2004; Lease and Hirsh, 2005). FlyBong involves lower amounts of animal handling than these approaches and consequently reduces both testing time and experimental error due to animal manipulation. We place flies into recording tubes 1 day before testing in order to habituate them to the environment, and they are then not moved or manipulated for the duration of the experiment. Here, we have shown that the drug concentration received by each is equivalent, as the amount of drug per tube is independent of recording tube location within the monitor, and does not vary significantly between experiments. Thus the behavioral variability that we see in sensitivity to acute cocaine exposure and locomotor sensitization to two repeated exposures, where response is present only in a fraction of the population, likely represents genetic and environmental influences that occur before testing. This therefore suggests that FlyBong can be used for the selective breeding of flies that are either sensitive and develop locomotor sensitization versus those that do not, in order to identify the potential genetic contributions for these behaviors, as an alternative to a genetic screen.

### FlyBong Identifies Cocaine-Specific Behavioral Phenotypes of Genetic Mutants

We have shown in several mutant strains that analysis of individual flies aids in differentiating cocaine-specific from non-specific effects on locomotor activity. We identify *fmn* mutant as hyperactive at the population level and with increased arousal in response to the control, warm airflow, in agreement with previously reported behavioral phenotypes of this mutant (Kume et al., 2005). Sensitivity to vCOC at individual level is not cocaine-specific, in agreement with previously published reports of a lack of sensitivity to cocaine administration in dopaminergic knockout mice (Chen et al., 1992; Giros et al., 1996; Mead et al., 2002) and with results of electrophysiological studies on the function of DAT in *Drosophila* (Makos et al., 2010). This shows that FlyBong can identify mutants that show baseline hyperactivity, and can furthermore distinguish between cocaine-specific versus non-specific arousal.

This is further exemplified with our results of increased individual sensitivity to warm air in *per^*01*^*, *cyc^*01*^*, *tim^*01*^* and *pdf^*01*^* flies. The increased sensitivity phenotype in *cyc^*01*^* and *per^*01*^*mutants is separate from their inability to develop locomotor sensitization. Both at the population level and at the individual level they do not develop locomotor sensitization to vCOC, nor do they develop locomotor sensitization to control, warm air. *Clk^Jrk^* mutant flies show cocaine-specific decrease in sensitivity, accordance with the inhibition of locomotor activity by light in *Clk^Jrk^* mutant flies (Lu et al., 2008), and inability to develop locomotor sensitization to vCOC. Thus, *per^*01*^*, *cyc^*01*^*, and *Clk^Jrk^* lack ability to modulate neuronal activity in response to repeated exposures to vCOC, which is in case of *Clk^Jrk^* accompanied with specific decrease in sensitivity. This shows separate molecular mechanisms involved in the regulation of behavioral phenotype to the acute versus multiple exposures of vCOC, which can be precisely measured using FlyBong.

Individual analysis identified an increased sensitivity to cocaine in *pdf^*01*^* and *tim^*01*^* mutants, which is in accordance with the role of *pdf* in regulating light-mediated arousal (Renn et al., 1999; Shimada et al., 2016) and promoting of locomotor activity by light in *tim^*01*^* mutants (Lu et al., 2008). However, locomotor sensitization to vCOC in *pdf^*01*^* and *tim^*01*^* flies is not cocaine-specific and will require further study in order to determine the role that these genes play in neuronal plasticity. Since both mutants have increased sensitivity to the warm air and develop locomotor sensitization to the warm air, future studies will have to use an alternate mode of delivering COC. Our results from population level analysis of *pdf^*01*^* show normal sensitivity to vCOC, similar to normal sensitivity measured as negative geotaxis of a population of *pdf^*01*^* mutants to vCOC (Tsai et al., 2004). However, our analysis of individual flies defined new phenotypes that were not immediately obvious in a population. Thus, analysis of the locomotor activity of individual flies, as well as their respective controls, significantly aids in differentiating cocaine-specific from non-specific effects. As many genetic mutations can have pleiotropic effect on arousal, individual analysis could aid in identifying currently unknown arousal phenotypes in other genetic mutants.

We have developed, characterized and validated FlyBong using environmental and genetic manipulations. In the future, Fly Bong can therefore be used to further dissect the genetic contribution to mechanisms that govern sensitivity, along with those that are involved in the processes of neuronal plasticity, which underlie locomotor sensitization. Statistical analysis of a population versus individual response further facilitates different approaches aimed at unbiased gene discovery, such as genetic screens or selective breeding. This will aid in understanding the mechanisms by which addictive drugs can change brain functioning and behavior not only in *Drosophila*, but in other laboratory animals and humans.

## Author Contributions

AF and RAW conceived the study, AF planned and performed experiments, analyzed the data and participated in interpreting the data and drafting the manuscript. SA participated in experiments and data analysis. RAW designed experiments, interpreted data and wrote the manuscript with input from AF. All authors read and approved the final manuscript.

## Conflict of Interest Statement

The authors declare that the research was conducted in the absence of any commercial or financial relationships that could be construed as a potential conflict of interest.
