# Supplementary material for: High Throughput Measurement of Locomotor Sensitization to Volatilized Cocaine in Drosophila melanogaster
Source: Front Mol Neurosci. 2018 Feb 5;11:25. doi: 10.3389/fnmol.2018.00025 (PMC5807336; doi:10.3389/fnmol.2018.00025)
Supplement: Supplementary file 1 [file Presentation_1.pdf]

Supplementary Materials:

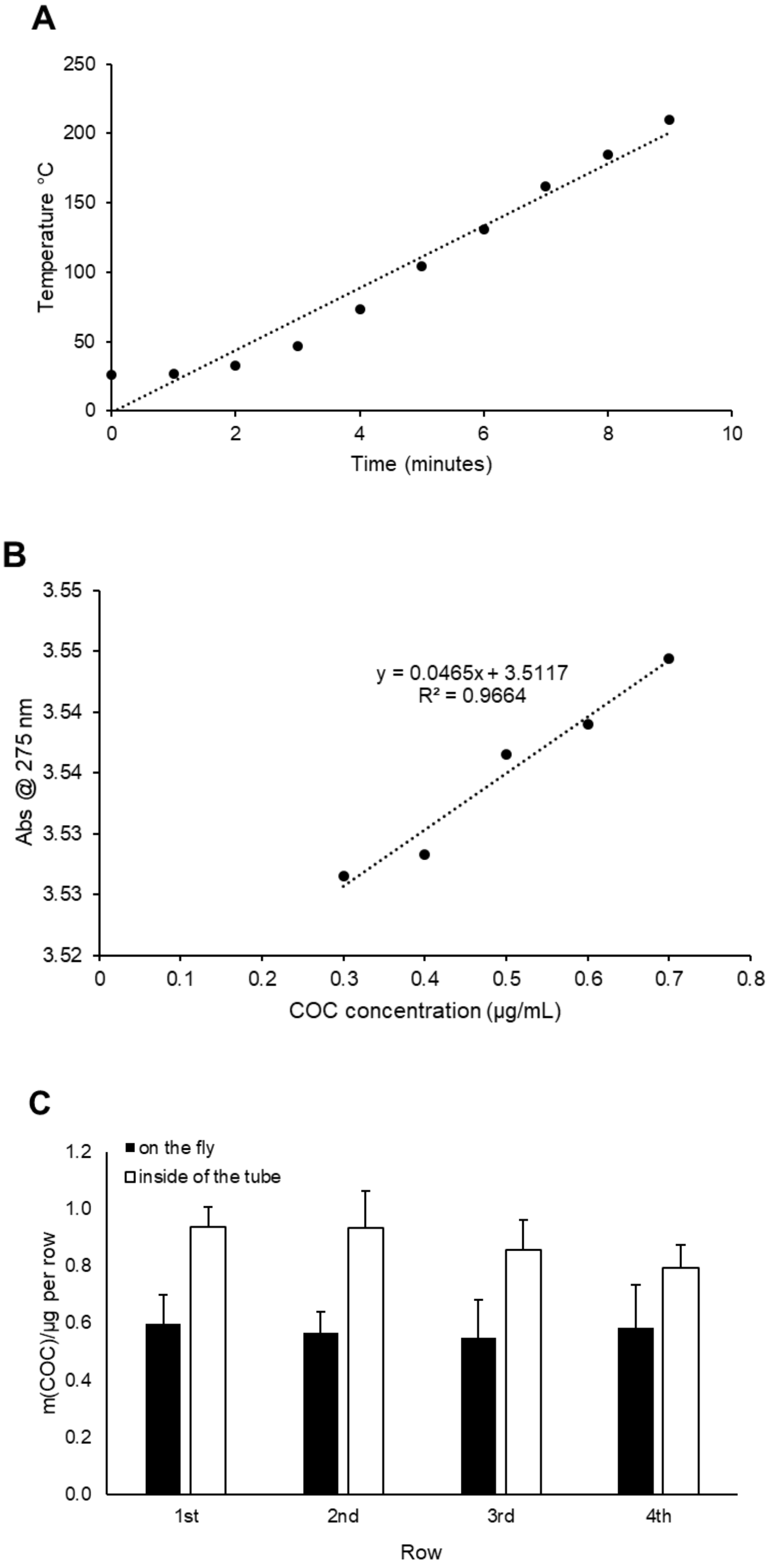

D

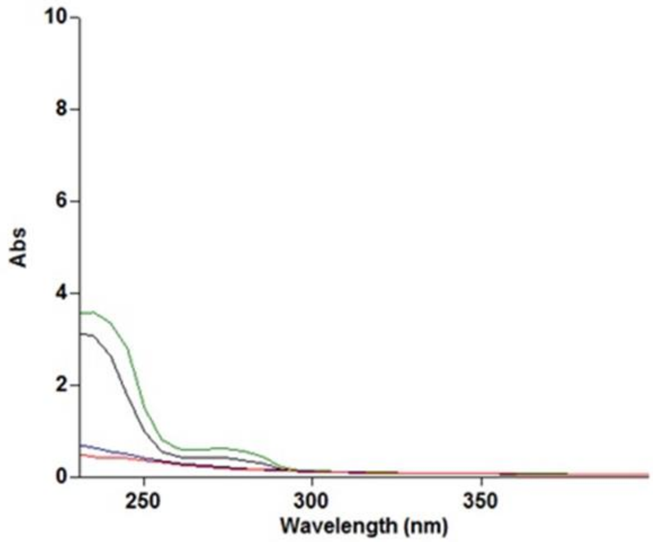

Figure 1. Optimization of volatilization temperature for cocaine (COC) delivery and measurement of COC on the fly and inside the DAM system tube. **A)** Volatilization of COC depends on the temperature of the flask and duration of heating. After 8 minutes, internal temperature reaches 185 °C, the known temperature of volatilization for the HCl form of COC. The standardized optimal heating duration for further experiments was chosen as 8 minutes. **B)** Concentration of COC in samples (on the flies and inside of tubes) was determined using COC calibration curve. Calibration curve was determined from COC standards prepared from cocaine-hydrochloride concentrations (≥97,5%) (Sigma Aldrich) by measuring their absorbance at 275 nm. We used a linear regression method to determine the slope of the line that we used to calculate COC concentration in each sample. **C)** COC is evenly distributed between all tubes in the DAM monitor and all flies. Mean amount of COC +SEM delivered to each row of the vertical DAM monitor, which consists of 32 slots (4 rows and 8 columns). **D)** UV-VIS spectra of COC in the range 400-230nm dissolved in distilled water using plastic cuvettes. Random samples after COC volatilization (blue and red) and COC standard of known concentration, 0.3 mg/mL (gray) and 0.5 mg/mL (green) have the same absorption peaks around 230 and 275 nm characteristic for COC maximum absorbance. Based on overlapping UV-VIS spectra of experimental samples (with heating), and standards samples (without heating) we conclude that COC remains stable after volatilization.

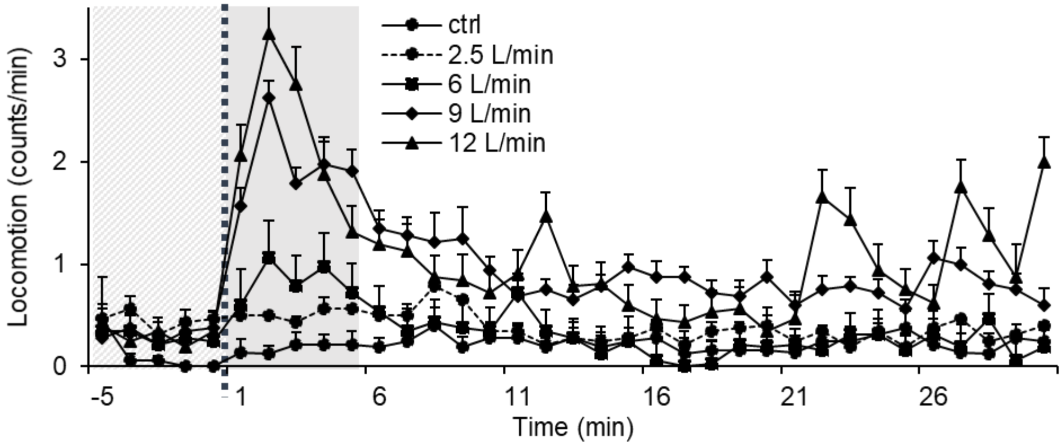

Figure 2. Level of locomotor activity in *wt* males depends on the strength of airflow. Locomotor activity was measured in the DAM system for flies exposed to different air flows (without COC or heating of the flask) for one minute. Kinetic graph shows average +SEM for 32 flies per group at a resolution of one minute. The light gray panel indicates the 5 minutes immediately prior to exposure, the dotted line is the time of exposure and the dark gray panel indicates 5 minutes after exposure. Increasing the air flow above 2.5 L/min leads to a transient and significant increase in locomotor activity. An air flow of 2.5 L/min was chosen as optimal for further experiments.

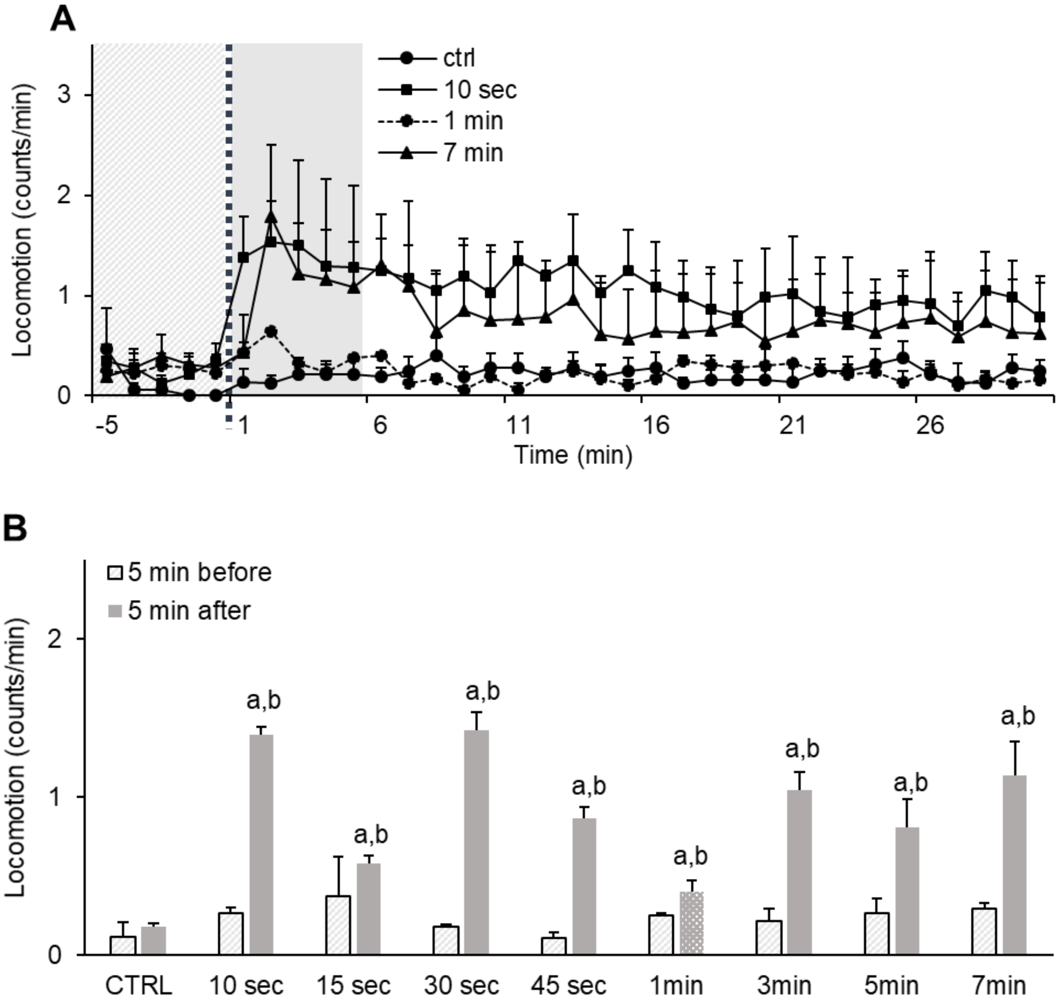

Figure 3. Effect of warm airflow duration on locomotor activity in *wt* males. **A)** Average activity + SEM for flies exposed to different duration of 2.5 L/min air flow from a flask heated for 8 minutes, without COC. Kinetic graph shows average +SEM for 32 flies per group at a resolution of one minute. The light gray panel indicates the 5 minutes immediately prior to exposure, the dotted line is the time of exposure and the dark gray panel indicates 5 minutes after exposure. **B)** Histogram of different durations of air flow plotted as mean population locomotor activity (32 flies per group), 5 minutes before and 5 minutes after exposure to warm air. A warm airflow of one minute leads to no significant difference between levels of activity before and after exposure, so this was chosen as the standard duration for COC delivery. Statistical significant differences ($p \leq 0.05$) are indicated by a: comparison of activity before and after exposure (within the group using t-test for dependent samples) and b: activity in the control group (no air flow) compared to after exposure in groups exposed to warm air flow (t-test for independent samples).

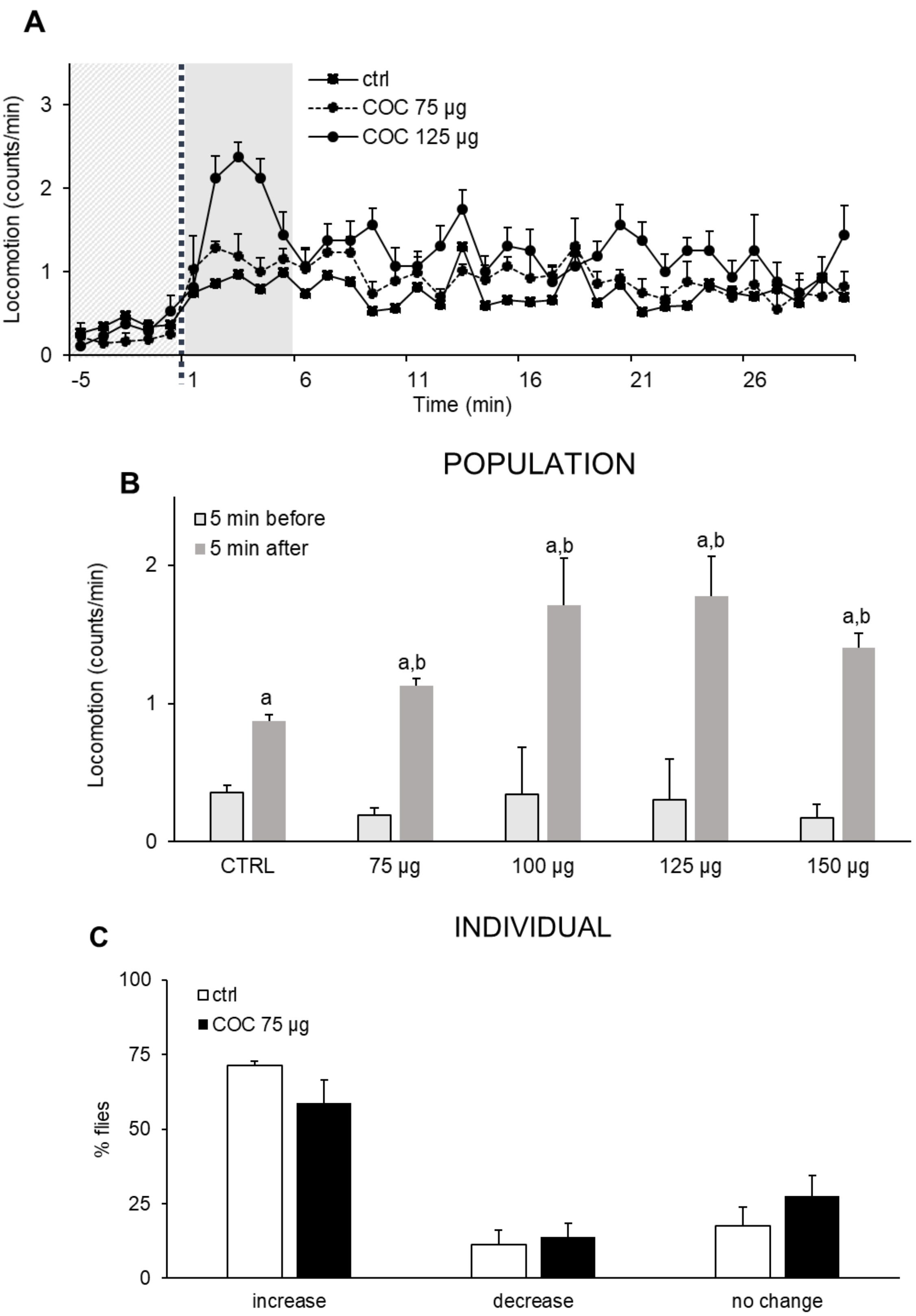
A
ctrl
COC 75 µg
COC 125 µg
Locomotion (counts/min)
0
1
2
3
-5
1
6
11
16
21
26
Time (min)
POPULATION
B
5 min before
5 min after
Locomotion (counts/min)
0
1
2
a
a,b
a,b
a,b
a,b
CTRL
75 µg
100 µg
125 µg
150 µg
INDIVIDUAL
C
ctrl
COC 75 µg
% flies
0
25
50
75
100
increase
decrease
no change

Figure 4. At the individual level, response of female *wt* flies is not COC specific. **A)** Kinetic graph of locomotion expressed as number of counts per minute for control group of flies (n=32, exposed to warm air), and test group exposed to 75 µg of volatilized COC (n=32), and 150 µg of volatilized COC (n=32). The light gray panel indicates the 5 minutes immediately prior to exposure, the dotted line is the time of exposure and the dark gray panel indicates 5 minutes after exposure. **B)** Histogram of different amounts of volatilized COC (75 to 150 µg) plotted as a mean of the population (32 flies) locomotor activity 5 minutes before and 5 minutes after exposure to COC. Statistical significance ($p \leq 0.05$): is indicated by: a: comparison of activity in the 5 minutes immediately before and after exposure (within the group using t-test for dependent samples); b: activity after exposure, compared between the control group and group exposed to volatilized COC (t-test for independent samples). **C)** Amount of individual fly locomotor activity 5 min before exposure was compared to 5 minute after exposure to 75 µg of volatilized cocaine (n=32) and categorized as an increase, decrease or no change and compared to control group (n=32) that received warm airflow. There is no difference in sensitivity between control group and group that received 75 µg of COC. Starting data were same as B) from which categories increase, decrease and the same were derived.

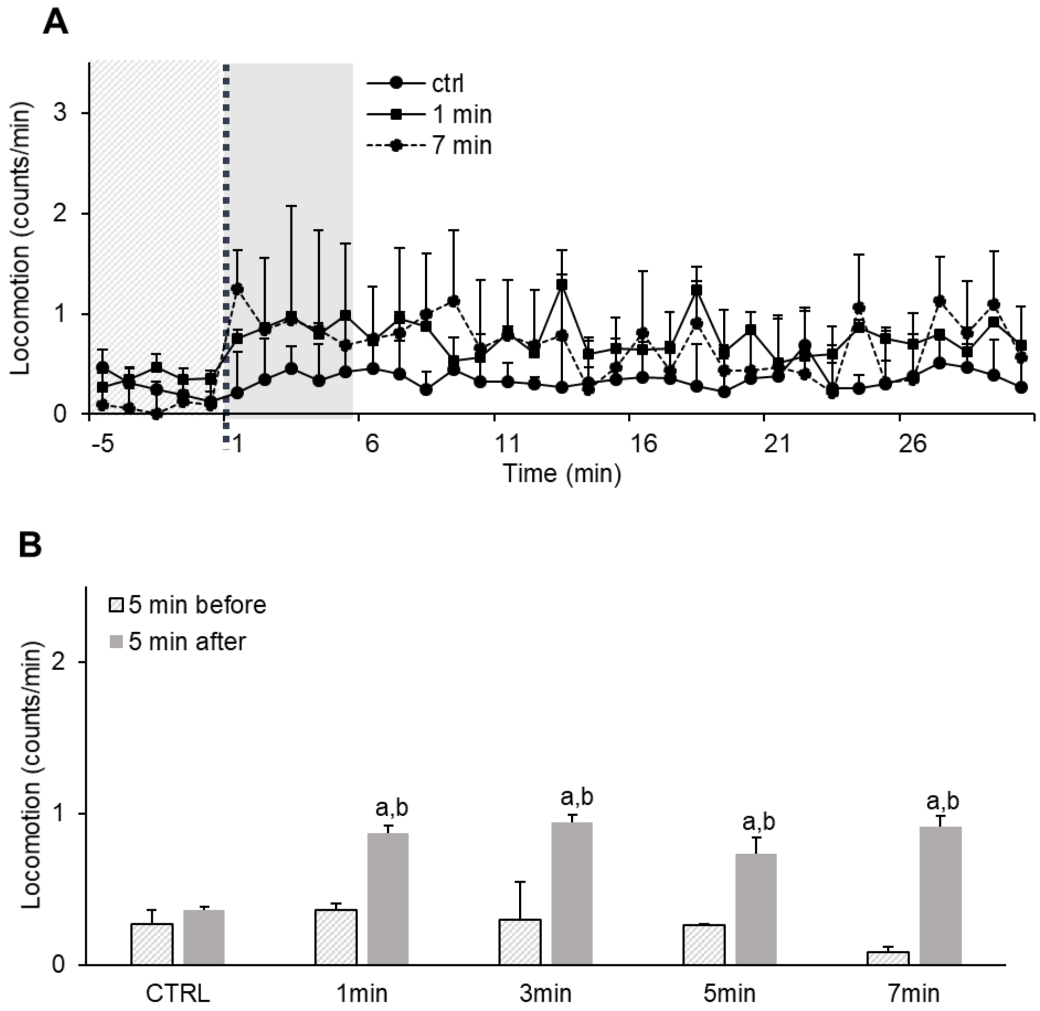

Figure 5. Effect of warm airflow duration on locomotor activity in *wt* females. **A)** Average activity + SEM for flies exposed to different duration of 2.5 L/min air flow from a flask heated for 8 minutes without COC. Locomotor activity was measured in DAM system at a resolution of one minute. Kinetic graph shows mean +SEM for 32 flies per group in the 5 minutes immediately before (light gray panel), and after exposure (dark gray panel). **B)** Histograms of locomotor activity for different durations of airflow plotted as population average (32 flies) 5 minutes before and 5 minutes after exposure to warm air. All tested durations of air flows lead to similar and statistically significant difference in locomotor activity before and after exposure to warm air. This includes the one minute duration which did not lead to a significant change compared to baseline in males. Statistical significance ($p \leq 0.05$) indicated for: a: within group comparison (t-test for dependent samples); b: between group comparison for the 5 minute period after warm air (t-test for independent samples).

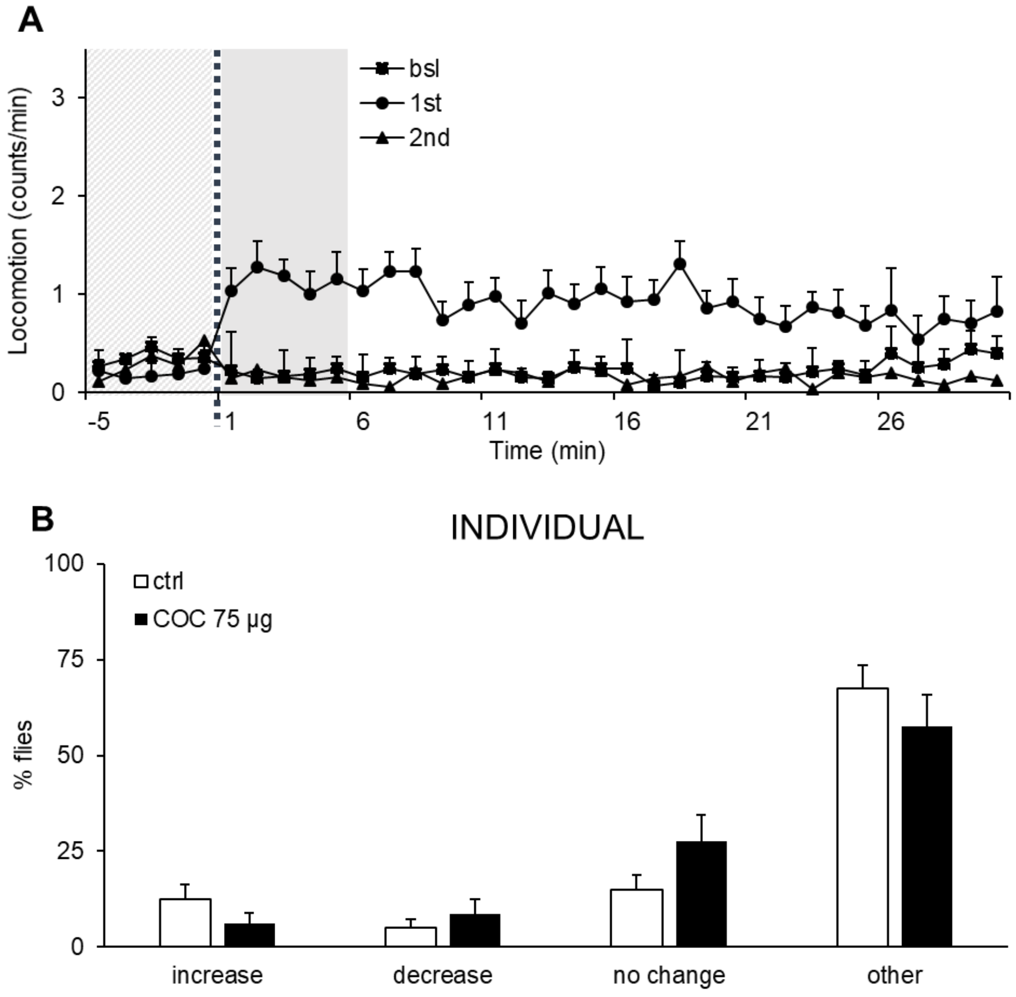

Figure 6. Our standard protocol for COC administration does not induce locomotor sensitization in *wt* female flies. A) Kinetic graph of locomotion expressed as number of counts per minute for group exposed to volatilized 75 µg COC (n=32), once at 09:00 AM (1st) and then at 03:00 PM (2nd), along with their baseline (bsl) activity before drug administration. The light gray panel indicates 5 minutes immediately prior to exposure, the dotted line is the time of exposure and the dark gray panel indicates 5 minutes after exposure. Locomotor activity after 2nd exposure is similar to baseline levels. **B)** Analysis of individual flies from data in A). Amount of individual fly locomotor activity 5 min before exposure was compared to 5 min after the first and second exposures to 75 µg of volatilized cocaine (n=32). These were categorized as an increase, decrease, no change or other (flies that did not satisfy criteria for previous three groups), and compared to the control group (n=32) that received warm airflow only. Percent of flies in each category is similar between group that received 75 µg of COC and control.

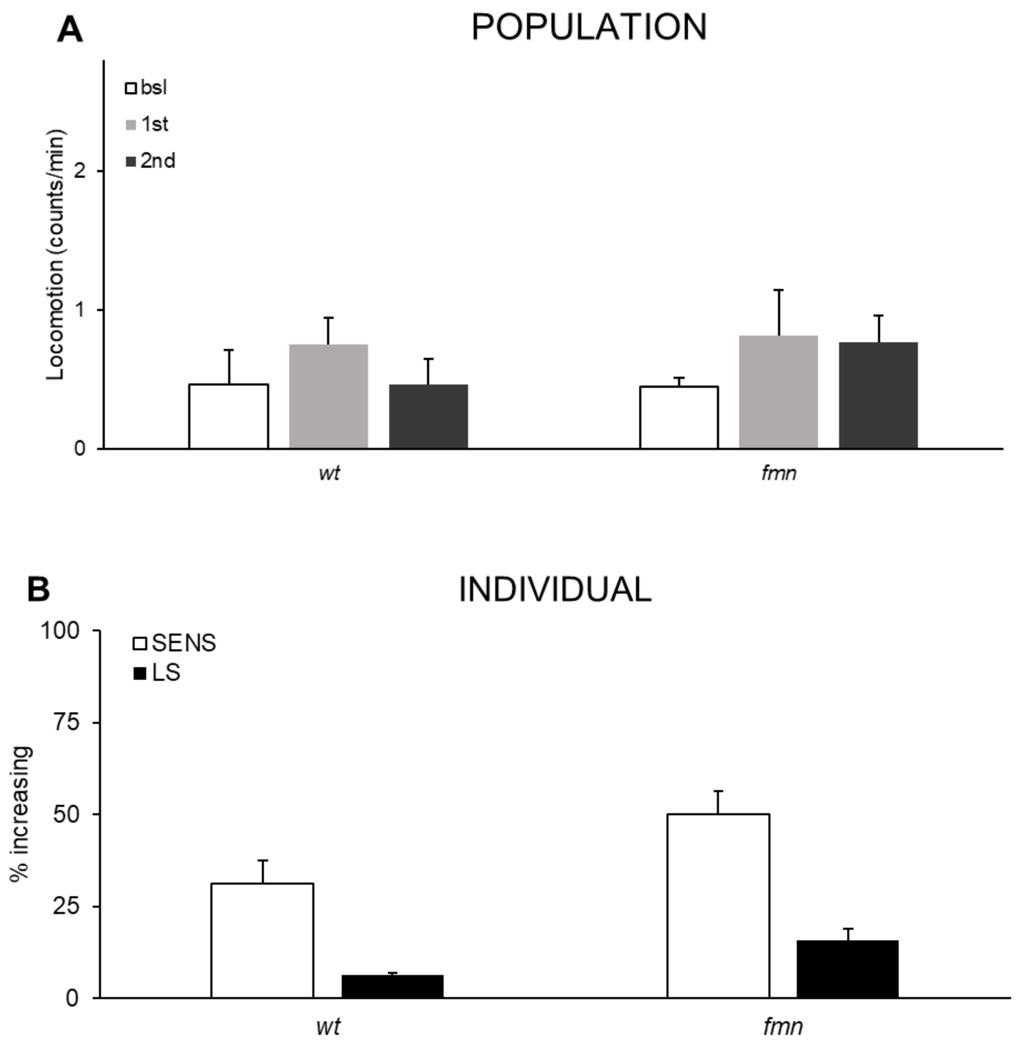

Figure 7. Influence of warm air flow on sensitivity and locomotor sensitization of *fmn* mutants. **A)** Average locomotor activity (counts/min) during baseline, 5 minutes before exposures (bsl), 5 minutes after first exposure (1st) and 5 minutes after second (2nd) exposure to warm air flow (2.5 L/min, for 1 minute after 8 minutes of heating) given 6 hours apart. Fly populations were either wild type (*wt*) or *fmn* mutants (n=32 for each group). **B)** Percentage of individual flies showing sensitivity (SENS) indicating increased locomotor activity to first exposure to warm air flow (2.5 L/min, for 1 minute after 8 minutes of heating) and flies showing further increase in locomotor activity to the second exposure (LS). Starting data were same as A) from which categories increase, decrease and same were derived.
